# Supplementary material for: Simulations approaching data: cortical slow waves in inferred models of the whole hemisphere of mouse
Source: Commun Biol. 2023 Mar 13;6:266. doi: 10.1038/s42003-023-04580-0 (PMC10011502; doi:10.1038/s42003-023-04580-0)
Supplement: Supplementary file 2 — Supplementary Information [file 42003_2023_4580_MOESM2_ESM.pdf]

Simulations Approaching Data: Cortical Slow Waves in Inferred Models of the Whole Hemisphere of Mouse

Cristiano Capone[1, *, **], Chiara De Luca[1, 2, *], Giulia De Bonis[1], Robin Gutzen[3,4], Irene Bernava[1], Elena Pastorelli[1], Francesco Simula[1], Cosimo Lupo[1], Leonardo Tonielli[1], Francesco Resta[5], Anna Letizia Allegra Mascaro[5,6], Francesco Pavone[5,7], Michael Denker[3], and Pier Stanislao Paolucci[1]

[1]INFN, Sezione di Roma, Rome, Italy
[2]PhD Program in Behavioural Neuroscience, "Sapienza" University of Rome, Rome, Italy
[3]Institute of Neuroscience and Medicine (INM-6) and Institute for Advanced Simulation (IAS-6) and JARA-Institute Brain Structure-Function Relationships (INM-10), Jülich Research Centre, Jülich, Germany
[4]Theoretical Systems Neurobiology, RWTH Aachen University, Aachen, Germany
[5]European Laboratory for Non-Linear Spectroscopy, Sesto Fiorentino, Italy
[6]Neuroscience Institute, National Research Council, Pisa,Italy
[7]University of Florence, Physics and Astronomy Department, Sesto Fiorentino, Italy
[*]These authors contributed equally to this work
[**]Corresponding Author: cristiano0capone@gmail.com

February 10, 2023

Supplementary Note 1

Variability across trials

To show the variability of the observed phenomena across different trials from the same mouse, in Fig. S1 we provided the experimental distributions of macroscopic observables of each of the six analysed trials for both analysed mice. The cumulative distributions of local velocities, directions and inter-wave intervals are depicted in panels **a** and **b** for mouse 1 and 2, respectively. Furthermore, we quantitatively compared these observables across trials evaluating the Earth Mover's Distance (EMD) for each couple of different trials from the same mouse, as reported in panels **c** and **d** for mouse 1 and 2, respectively.

Thanks to the high spatial resolution of the dataset, this variability can be easily detected and quantified. Such differences may be due to a change in the experimental conditions (i.e., variation of the anaesthesia level).

Inferring neuro-modulation parameters through likelihood maximization

It is technically possible to include the oscillatory neuro-modulation contribution in the likelihood and to infer the related parameters by likelihood maximization. However, we observed that the inferred parameters define a generative model that does not fully capture the complexity of the dynamics observed in the data (see Fig. S2, panels **a** and **b** for mouse 1 and panels **e** and **f** for mouse 2). Namely, in contrast with the two-step approach we proposed (inner+outer loop), the single inference step including the neuro-modulation

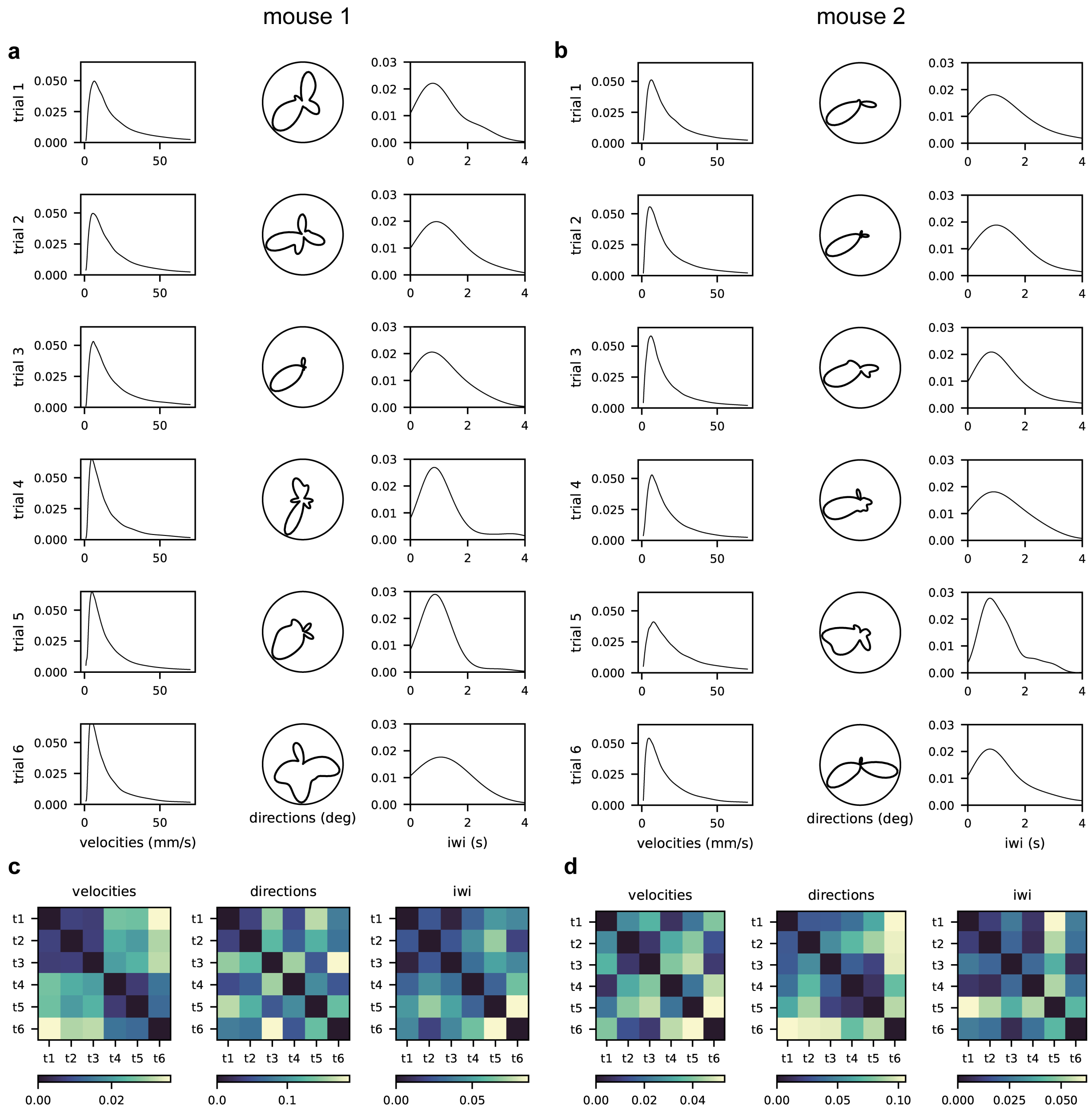

Figure S1: **Summary of wave propagation properties in experimental trials for the two analysed mice.** Measures for the quantitative characterization and comparison of waves cumulated across all pixels in each trial in mouse 1 (left) and 2 (right), respectively. **a/b**. Cumulative distributions of local wave velocities, directions and inter-wave intervals for each trial and each mouse. **c/d**. EMD between the macroscopic distributions of the same observable from different trials of the same mouse, for each of the two mice.

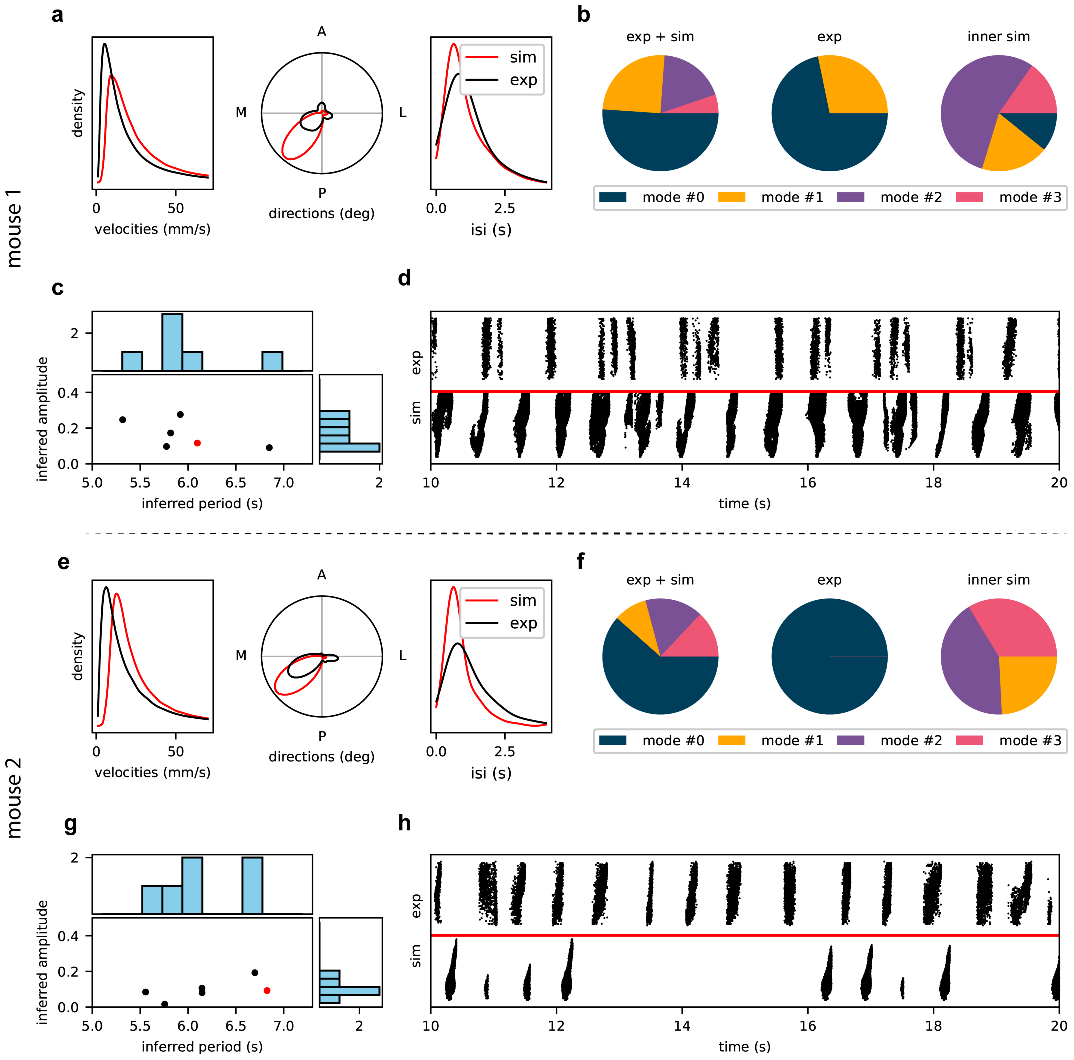

Figure S2: **Inferring neuro-modulation parameters through likelihood maximization. a/e**. Comparison of global metrics (waves velocities, directions and IWI) for data and simulation, when neuromodulation parameters are inferred through likelihood maximization. **b/f**. GMM analysis to identify propagation modes. Comparison between experimental data and simulations. **c/g**. Histograms and scatter plot of amplitude and period neuro-modulation parameters inferred by likelihood maximization for each of the six trials. **d/h**. Rastergram of waves onset, showing the comparison of the dynamics between model and data for a single trial (red point in panels C/G.).

contribution does not reproduce the temporal non-stationarity observed in data (in particular, in the IWI distribution).

Inferred values for neuro-modulation amplitude and period are separately reported for each trial and mouse, panels **c** and **g**, showing a good consistence between different temporal recordings (chunks). We also reported a direct comparison between the temporal dynamics of the data and the model for each of the two mice, panels **d** and **h**.

Our interpretation is that direct inference in the inner loop of the extra parameters for neuro-modulation is not trivial. One reason may e.g. be that it is necessary to infer the correct phase, but being the signal noisy, a larger amount of data would in fact be required.

A posteriori validation of the simulated wave modes

The aim of this Section is to quantitatively assess the statistical stability of clustering results through GMM. Corresponding results are depicted in Fig. S3.

In the top row of panels S3**a** and S3**c**, we report the 3 modes of propagation identified on the two mice through the GMM. When clustering at the same time experimental and simulated wave, instead, 4 main modes of propagation are recognized by GMM (middle row in panels S3**a** and S3**c**).

As a control, then, we replicated the same procedure on the dataset composed of experimental waves and the corresponding "shuffled" simulated waves, where the latter are obtained through a channel permutation (equal for all the simulated waves). We get again 4 modes, bottom row of panels S3**a** and S3**c**. As expected, the resulting propagation modes are different from those obtained when analyzing the experimental + simulated dataset.

In the leftmost column of panels S3**b** and S3**d** are reported the fraction of waves falling into each propagation mode, for each dataset analyzed (experimental, experimental+simulated, experimental + shuffled) and each mouse.

As a first validation analysis of the fidelity of the model, we forced the optimal simulation and the control (shuffled) simulation over the empirical (experiment-only fitted) modes. The fraction of detected modes is represented in top row of panels S3**b** and S3**d**. We also measured how well these modes describe the simulated datasets. To do so, we split the experimental dataset in a training and a testing dataset, we fit the GMM over the training one only (75% of waves) and measured the log-likelihood associated with the classification of the testing experimental dataset (upper bound), the simulated waves datasets and the shuffled control dataset (lower bound), as reported in Table 1. The confidence in the representation of the simulated dataset is $88 \pm 2\%$ (mouse 1) and $85 \pm 1\%$ (mouse 2).

Looking at the occupancy of each of 4 modes obtained when clustering experimental and simulated waves together (second row, left), and comparing it with the occupancies of experimental waves and simulated waves separately, it is worth noting that, for both mice, the three main modes identified are present in both data and simulation, whereas the fourth mode is less frequent and mostly present only among simulated waves.

At variance, when comparing the occupancies of experimental waves and shuffled simulated waves taken alone — clustered into the 4 modes obtained from the joint experimental + shuffled dataset —, a net separation between the modes can be appreciated. E.g., most of experimental waves are cathegorized into mode #0, absent among control waves.

Table 1: Log-likelihood associated to the classification of the testing experimental dataset (upper bound), the simulated waves datasets and the shuffled control dataset (lower bound) by GMM modes fitted over a portion of the experimental dataset. Errors are computed over 20 different splittings of the experimental dataset.

	Log-likelihood			
mouse	experimental dataset	simulated dataset	shuffled dataset	confidence
n. 1	91 ± 1	-121 ± 2	-1720 ± 8	88 ± 2 %
n. 2	138 ± 2	-17 ± 1	-903 ± 4	85 ± 1 %

Detecting the number of components in the GMM

The selection of the number of components in the GMM model is automatically determined using the Bayes Information Criterion (BIC) [1], relying on a likelihood maximization protocol. This criterion provides an estimate for the goodness of the fit made by the GMM in terms of predicting the data we actually have. The lower is the BIC score, the better is the model to actually predict the available data (and, by extension, the true unknown distribution of all data). Specifically, the optimal number of Gaussian components to be considered is thus defined as the minimum one producing a plateau in the BIC gradient, as shown in Fig. S4.

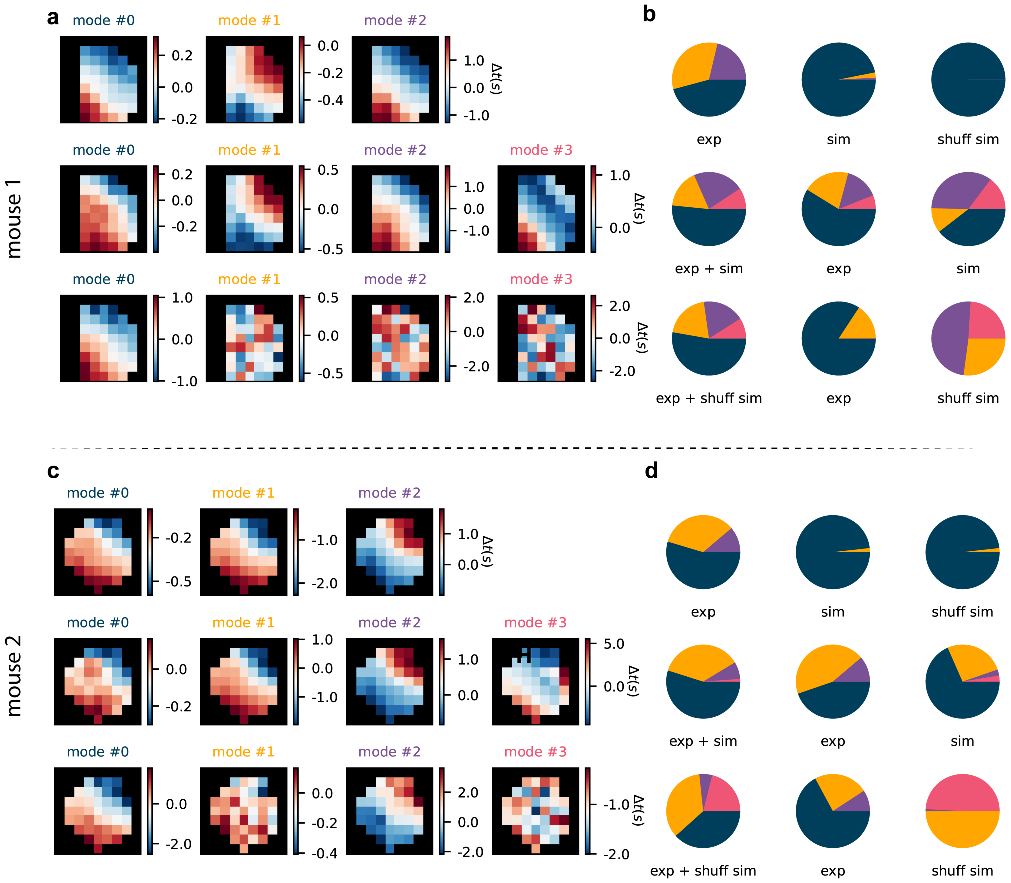

Figure S3: ***A posteriori* validation of simulated wave modes.** For each of the two mice, the optimal simulation is compared with a control case (shuffled simulation), obtained by the same permutation of channels for all the waves. **a/c**. Wave propagation modes identified by GMM fitted on experimental waves only (first row, 3 modes identified), on both experimental and simulated waves (second row, 4 modes identified), and on both experimental and shuffled simulated waves (third row, 4 modes identified). **b/d**. Classification of waves (see bottom label in each plot) according to the GMM modes detected in panels **a** and **c**: 3 modes (detected on experimental waves) for the first row, 4 modes for the second row (detected on experimental + simulated waves), and 4 modes for the third row (detected on experimental + shuffled simulated waves).

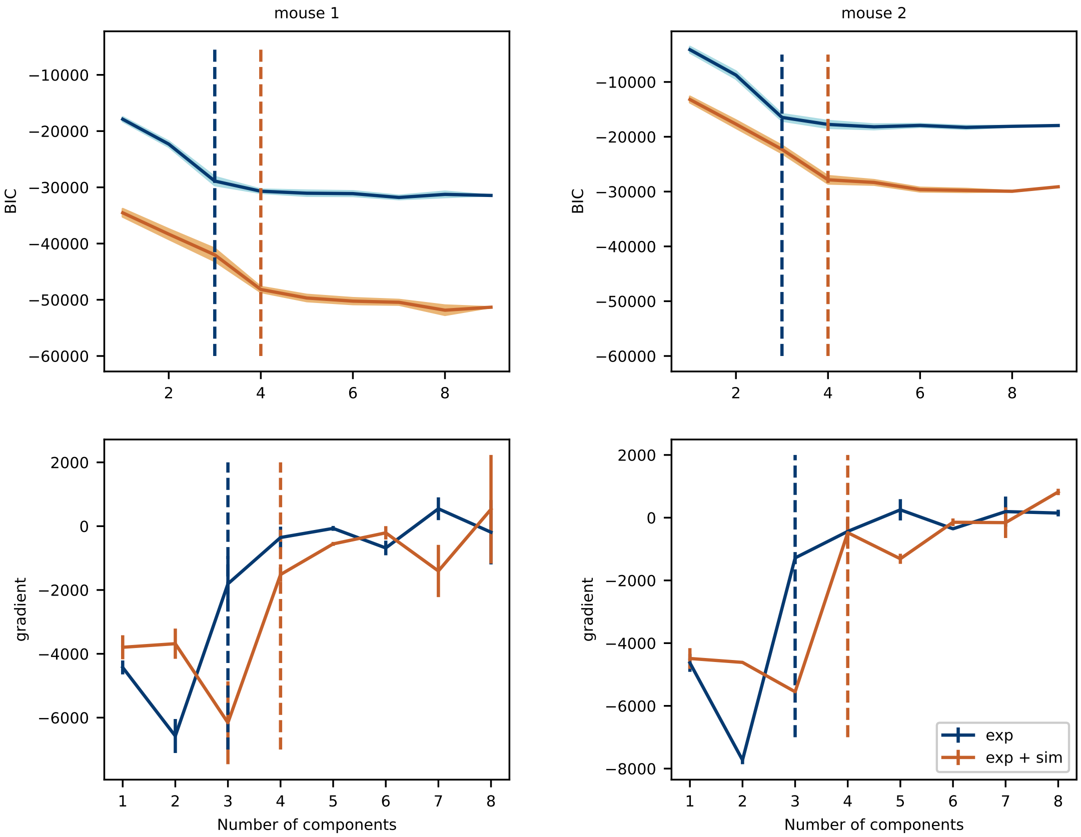

Figure S4: **Number of GMM components identified by the BIC criterion.** *Upper row*: BIC score for the GMM method when fitting different numbers of components, for experimental data (blue) and experimental+simulated data (orange). *Lower row*: gradient of the BIC score from corresponding panels above. Left panels refer to mouse 1, right panels to mouse 2. Vertical dotted lines depict the optimal numbers of components identified for both datasets.

Simulation with pulse stimulation

It is important to note that the same network in the brain can generate different states with their specific propagation patterns and rhythms. Moreover, the brain response to "external" (e.g. sensory) stimulations strongly varies among different brain states. The building of data-driven realistic models, integrating such features, is an open challenge.

As a first step, we investigated the response to focal stimulation in different brain states (i.e. with different levels of neuro-modulation: low, medium, and high excitability, respectively), as reported in Fig. S5. We observe that when the excitability is low, the evoked wave does not propagate at all (top panel), while when the excitability is high, a global traveling wave is generated (bottom panel). Interestingly, an intermediate level of excitability allows for the propagation of a non-trivial wave pattern (middle panel).

Even if the connectivity of the model was inferred from a single brain state, the model supported the emergence of a rich dynamic repertoire of spatio-temporal propagation patterns, from those corresponding to deepest levels of anesthesia (spirals to classical postero-anterior and rostro-caudal waves) up to the transition to asynchronous activity, with the dissolution of the slow-wave patterns.

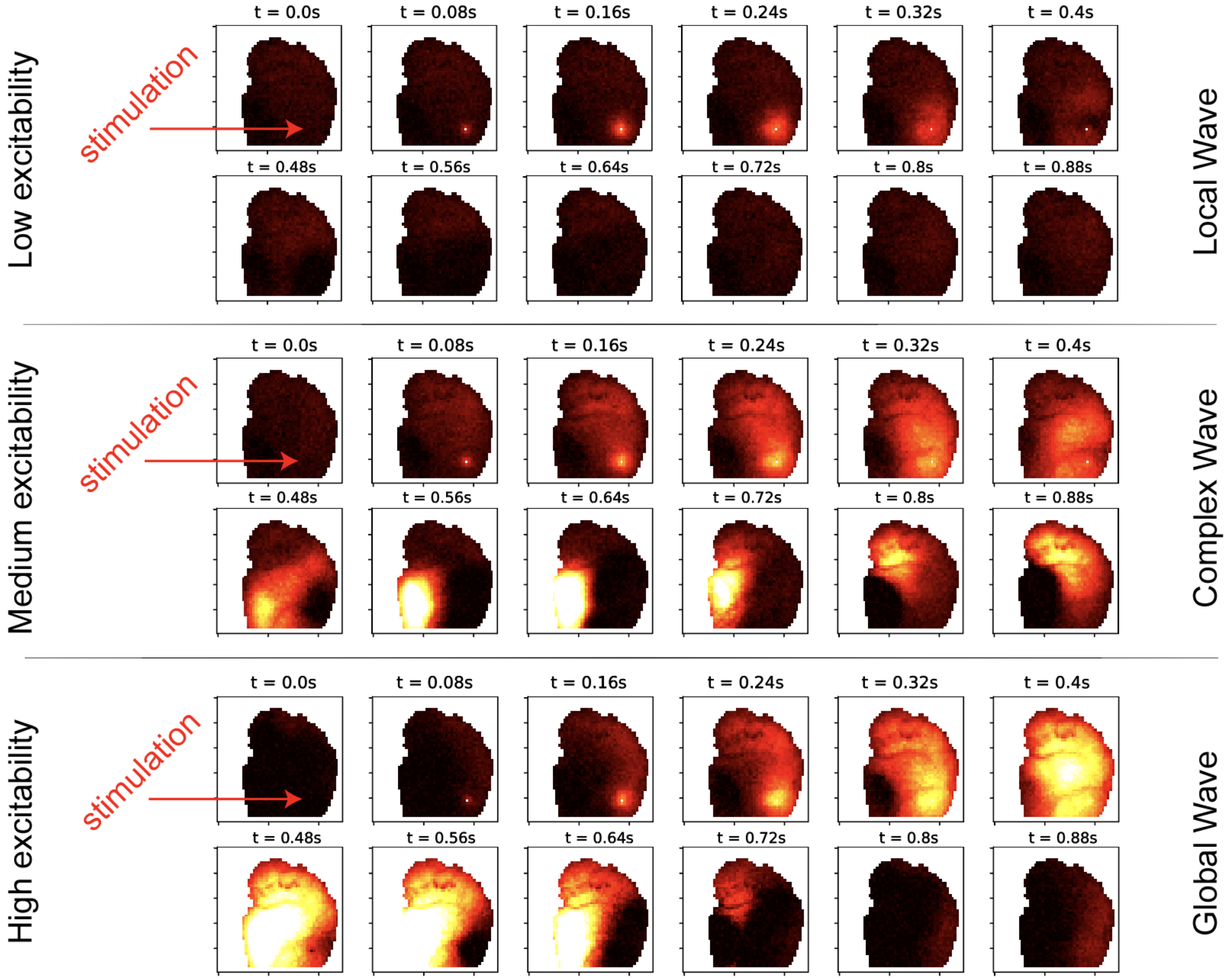

Figure S5: **Response to a focal stimulation in different brain states.** Different levels of neuromodulation (low, medium and high excitability, from top to bottom panel, respectively) have been used to simulate the different brain states. When the excitability is low, the evoked wave does not propagate (top panel), while when the excitability is high a global wave is generated (bottom panel). Interestingly, an intermediate level of excitability allows for the propagation of a non-trivial wave pattern (middle panel).

Non-stationary dynamics

The non-stationarity observed in the data, and reproduced in the model thanks to the neuro-modulation, can be easily visualized in the spectrograms reported in panel **a** of Fig. S6. While appreciable in both experimental and simulated (inner+outer loop) data, such non-stationarity is not observed in the spectrum generated by the inner loop solely. For a quantitative assessment, we computed the temporal auto-correlation of each frequency in the three different scenarios. In panel **b**, we show the normalized mean auto-correlation for the first 10 s for experimental data (left), the output of the inner loop (centre), and the output of the outer loop (right). The correlation rapidly decreases in time for both experimental data and simulation, whereas it remains high for the output of the inner loop, showing a stationary behavior. Moreover, these observations are shown to be consistent both inter-trial and inter-individual.

Spectrograms have been computed over a window of 4 s (100 data points) with 1 s of overlap. Then, each

frequency component v is auto-correlated in time according to the equation:

$$c_k = \frac{\sum_n v_n \, v_{n+k}}{\sum_n v_n \, v_n}$$

Finally, the mean of these coefficients $\{c_k\}$ for each time-bin is computed.

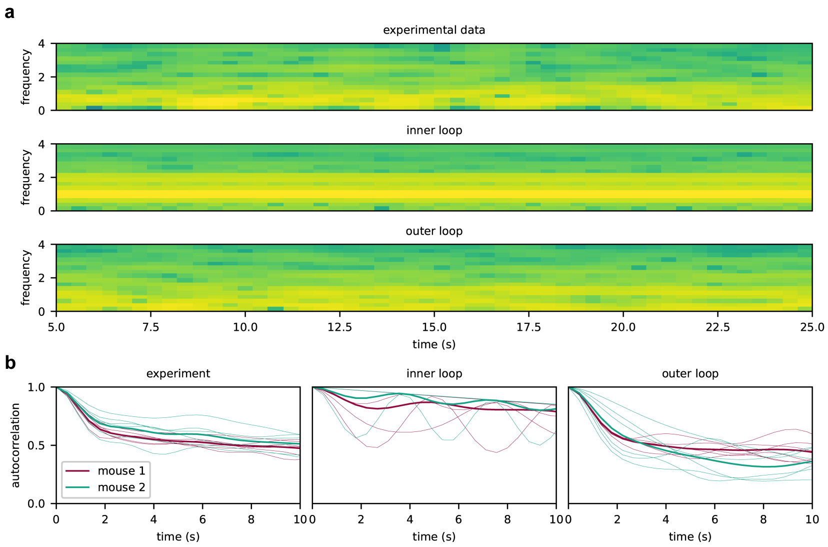

Figure S6: **Non-stationary dynamics.** **a**. Spectrograms for the average activity in a single trial from mouse 1, computed on experimental data (top) and simulation outcome (middle, inner loop only; bottom, outer loop). **b**. Normalized mean temporal auto-correlation of frequencies for both mice in experimental data (left) and simulated data (center, inner loop only; right, outer loop).

Acknowledgments

This work has been supported by the European Union Horizon 2020 Research and Innovation program under the FET Flagship Human Brain Project (grant agreement SGA3 n. 945539 and grant agreement SGA2 n. 785907) and by the INFN APE Parallel/Distributed Computing laboratory.

Supplementary References

[1] Gerda Claeskens and Nils Lid Hjort. *Model Selection and Model Averaging*. Cambridge University Press, 2008. URL: https://EconPapers.repec.org/RePEc:cup:cbooks:9780521852258.
